# A Rare Case of Neural Epidermal Growth Factor-Like 1 Protein (NELL-1) Antigen-Associated Membranous Nephropathy

**DOI:** 10.7759/cureus.34713

**Published:** 2023-02-07

**Authors:** Mohamed Zakee Mohamed Jiffry, Kristen Pitts, Meha Munir, Aimal Khan, Meagan Josephs

**Affiliations:** 1 Internal Medicine, Danbury Hospital, Danbury, USA; 2 Medicine, American University of the Caribbean School of Medicine, Cupecoy, SXM

**Keywords:** autoimmune disorder, general internal medicine, nell1, primary membranous nephropathy, general nephrology

## Abstract

Membranous nephropathy (MN) is an autoimmune disease resulting in nephrotic syndrome. Neural epidermal growth factor-like 1 protein (NELL-1) has been shown to cause a rare form of MN that is more likely to be associated with malignancy. We present a case of a 73-year-old female who was found to have a NELL-1-associated segmental MN. She presented complaining of generalized weakness, chills, and poor appetite, worsening over a one-week duration. Her kidney functions were noted to be markedly deranged, with a computed tomography scan of the abdomen showing evidence of chronic kidney disease. Further testing confirmed heavy proteinuria, although the etiology was still uncertain. A kidney biopsy revealed granular subepithelial immunoglobulin G deposits with subsequent immunohistochemical staining for NELL-1 antigen being positive. She improved with supportive care over the next few days. Despite an extensive workup, no underlying malignancy was found. NELL-1 is a rare yet recognized antigen target for the development of MN. Up to a third of patients with NELL-1-associated MN have associated cancer, thus requiring evaluation for underlying malignancy in this cohort.

## Introduction

Membranous nephropathy (MN) is an autoimmune disease resulting in a nephrotic syndrome that can be seen in adults worldwide. This condition results from antibodies that bind to antigens and form immune complexes. These immune complexes then deposit in the glomerular basement membrane (GBM), disrupting its function, and ultimately leading to proteinuria and renal failure.

In most cases of MN, the target antigen has been proven to be a phospholipase A2 receptor (PLA2R) [1]. In recent years, however, the utilization of mass spectrometry and immunohistochemistry have identified another antigen, neural epidermal growth factor-like 1 protein (NELL-1), that has been shown to cause a rare form of MN [2]. NELL-1-associated MN is more likely to be associated with malignancy.

We present a case of a 73-year-old female who was found to have a NELL-1-associated segmental MN.

## Case presentation

A 73-year-old woman presented to emergency services complaining of generalized weakness, chills, and poor appetite, worsening over the past week's duration. She also had intermittent episodes of nausea without vomiting. She had some subjective shortness of breath but no chest pain, overt dyspnea, or productive cough. She did not report urinary symptoms at this presentation such as dysuria, hesitancy, urgency, or incontinence of urine. She denied bowel symptoms such as diarrhea or constipation and denied visual disturbances or headaches.

Vital signs were remarkable for a mild fever of 37.8 °C, a pulse rate of 84 beats per minute, respiration of 17 breaths per minute, and blood pressure of 150/80 mmHg. A general examination revealed the woman who appeared her stated age in no acute distress. No appreciable lymphadenopathy was found. The cardiac examination revealed a regular rate and rhythm without murmurs. A respiratory examination revealed clear lung fields without audible wheezes or crackles. The abdomen was soft and nontender without suprapubic or flank tenderness. Another system examination was unremarkable.

She recently completed a course of cefuroxime for urinary tract infection (UTI) with her primary care provider and has had multiple prior UTIs in the past, with her past urine cultures growing Klebsiella pneumoniae and Enterococcus faecalis resistant to fluoroquinolones and tetracyclines. Her past medical history was otherwise significant for type 2 diabetes mellitus, heart failure with preserved ejection fraction, hypertension, hyperlipidemia, and post-ablative hypothyroidism. She denied a family history of kidney disease. She has a history of severe allergies to sulfa drugs.

Initial chemistry was remarkable for grossly elevated creatinine and blood urea nitrogen, with metabolic acidosis. A complete blood count showed normocytic anemia. A urinalysis revealed 2+ proteinuria. Initial investigative results are detailed in Table 1.

**Table 1:** Detailed initial laboratory investigations. ^*^Abnormal results have been identified in this column. ^**^Laboratory-specific reference ranges. eGFR, estimated glomerular filtration rate; CKD-EPI, chronic kidney disease epidemiology collaboration; BUN, blood urea nitrogen; WBC, white blood cell; RBC, red blood cell

Laboratory investigation (units)	Value (high/low values identified)^*^	Reference range^**^
Serum chemistry		
Serum creatinine (mg/dL)	5.28 (high)	0.5-1.04
eGFR per CKD-EPI 2021 (mL/minute per 1.73 m^2^)	8 (low)	≥60
Bicarbonate (mmol/L)	18 (low)	22-29
BUN (mg/dL)	72 (high)	6-23
Sodium (mmol/L)	139	135-145
Potassium (mmol/L)	4.7	3.5-5.3
Chloride (mmol/L)	106	97-107
Anion gap (mmol/L)	15	10-19
Lactic acid (mmol/l)	0.9	0.5-2.2
Magnesium (mg/dL)	2.2	1.6-2.6
Hemoglobin A1c	8.3	5.7-6.4
Complete blood count		
WBC (×10^9^ L^-1^)	8.5	3.5-10.0
Hemoglobin (g/dL)	10.0 (low)	12.0-16.0
Platelets (×10^9^ L^-1^)	296	150-400
Mean corpuscular volume (fL)	92.6	80.0-99.0
Mean corpuscular hemoglobin concentration (g/dL)	31.9	31.0-36.0
Urinalysis		
Appearance	Clear	-
pH	6.0	4.8-8.0
Specific gravity	1.010	1.001-1.035
Protein	2+ (high)	Negative
Blood	Trace	Negative
RBCs	0-2	0-2
WBCs	3-9	0-2
Leukocyte esterase	Negative	Negative
Bacteria/μL	3	0-1,359

**Figure 1:**
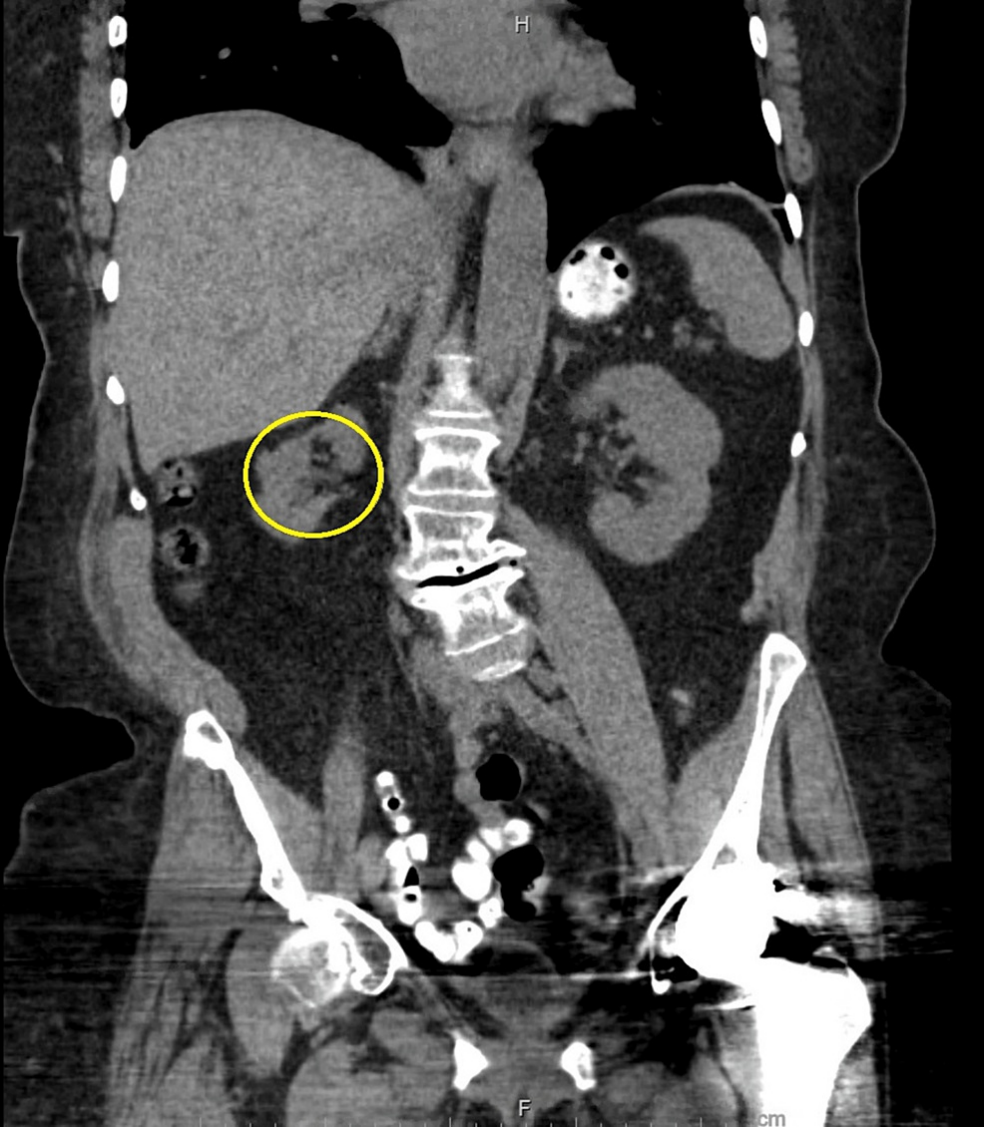
CT scan of the abdomen. *Yellow circle*: Right kidney is atrophic with chronic and irregular thickening of the parenchyma noted. CT, computed tomography

A chest X-ray did not show evidence of any acute cardiopulmonary process. A computed tomography scan of the abdomen and pelvis without contrast showed chronic irregular thickening of the right kidney attributed to her history of past urinary infections, without suspicious renal masses, stones, or evidence of obstructive uropathy (Figure 1).

Further workup of her acute on chronic kidney injury was ordered to evaluate for an etiology of her renal dysfunction. Heavy proteinuria was noted with an elevated urine protein: creatinine ratio and elevated 24-hour urinary protein. Preliminary serologic tests for etiologic determination were negative, as outlined in Table 2. To diagnose the cause of her renal failure, the patient was scheduled to undergo a kidney biopsy.

**Table 2:** Detailed subsequent laboratory investigations, including serologic tests for etiologic determination. Anti-GBM Ab, anti-glomerular basement membrane antibody; c-ANCA, anti-proteinase 3 antibody; p-ANCA, anti-myeloperoxidase antibody; AI, antibody index

Laboratory investigation (units)	Value (high/low values identified)	Reference range
Urinary studies		
Protein:creatinine ratio (mg/mg Cr)	2.95 (high)	≤0.16
24-hour protein (mg/24 hours)	2,143 (high)	≤160
Serologic tests		
Antinuclear antibody titer	Negative	Negative
C3 complement (mg/dL)	166	75-180
C4 complement (mg/dL)	33	10-40
Anti-GBM Ab (AI)	<0.2	0-0.9
c-ANCA (AI)	<0.2	0-0.9
p-ANCA (AI)	<0.2	0-0.9
Protein electrophoresis	No abnormal discrete bands suggestive of monoclonal gammopathy	-

**Figure 2:**
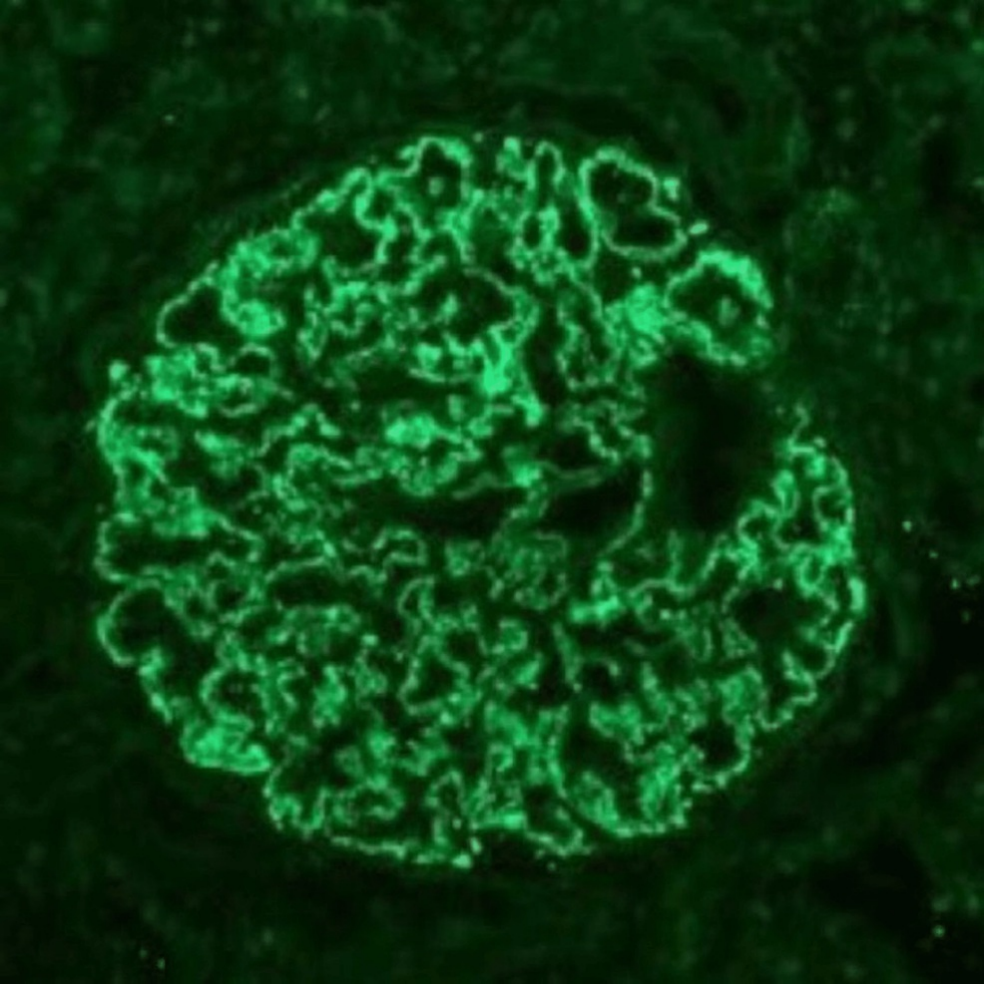
Immunofluorescence histology for IgG displays 3+ granular segmental subepithelial staining.

Under intermittent CT guidance, a 17-gauge Vim-Silverman needle (V. Mueller®, Device Technologies, Mulgrave VIC, Australia) was advanced into the lower pole of the left kidney, and four core biopsy samples were obtained, with no procedural complications. The left kidney was preferentially chosen as it made for anatomically easier access. The specimen was sent for pathologic analysis. Immunofluorescence findings of 3+ granular segmental subepithelial staining for IgG and 1+ for IgM, kappa, and lambda supported a diagnosis of segmental membranous nephropathy (Figure 2). Immunohistochemical stain for NELL-1 was positive in the segmental subepithelial deposits, supporting a diagnosis of NELL-1-associated segmental MN.

The patient’s management was supportive throughout her hospitalization. There was a gradual downtrend in her creatinine level over the next few days with intravenous (IV) fluid administration. A bicarbonate drip was given in tandem with the management of her metabolic acidosis. Blood pressure control was optimized with a multidrug regimen including doxazosin, labetalol, and lisinopril. She was discharged after recovery from her symptoms with a scheduled outpatient nephrology follow-up.

She was also scheduled for upper and lower endoscopies for malignancy workups that were unrevealing for evidence of malignancy. A high-resolution CT chest was done for an incidental lung nodule, which showed a stable indeterminate right middle lobe nodule measuring 4 mm, with a recommendation for six monthly CT surveillance.

## Discussion

MN is a rare autoimmune disease, in which circulating autoantibodies against podocyte antigens attack the glomerulus. It accounts for about 20% of cases of nephrotic syndrome in adults and is the most common cause of nephrotic syndrome in Caucasian adults. It is characterized by the accumulation of electron-dense subepithelial immune deposits and the formation of antigen-antibody complexes. In approximately 70% to 80% of cases of MN, the target antigen has proved to be a PLA2R, and in approximately 1% to 5% of cases, the target antigen has proved to be thrombospondin type-1 domain-containing 7A (THSD7A) [1], both of which are normally expressed podocyte antigens.

NELL-1 has been shown to cause a rare form of MN and is more likely to be associated with malignancy compared to the most common PLA2R and THSD7A subtypes, and NELL-1-positive MN was associated with a higher prevalence in individuals with underlying malignancy [2]. A single-center prevalence study determined that up to 8.9% of PLA2R/THSD7A-negative, nonlupus MN was due to NELL-1-associated MN [3].

Although the most commonly identified secondary causes of MN are autoimmune diseases, e.g., systemic lupus erythematosus (SLE), the second most commonly implicated was a malignancy. Various other secondary causes have been identified, including infections, graft versus host disease, allergic reactions, and drug reactions. An interesting study reported a possible association between mercury and NELL-1-associated MN [4]. NELL-1-associated MN has also been reported in patients with HIV infection [5].

Although the association between NELL-1 and malignancy remains ill-defined, a study on hypermethylation of the promoter site for the *NELL-1* gene showed an association with states of distant metastasis in patients with renal cell carcinoma, which may make it a potential prognostic epigenetic biomarker in this patient population [6].

A case review of patients with NELL-1-associated MN outlined that patients with NELL-1-associated MN who also had malignancy were significantly older than patients without malignancy, and up to 33% of these patients were found to have an underlying malignancy. Types of cancer that were found associated with NELL-1-associated MN included epidermoid lung cancer, metastatic pancreatic carcinoma, metastatic breast cancer, and infiltrating urothelial carcinoma. Additionally, the most common histopathologic parameter identified in kidney biopsies of NELL-1-associated MN was segmental with incomplete capillary loop staining at over 93% of identified cases, and IgG positivity was universally present [7]. Both these preceding findings were present in our case.

In the acute setting, supportive management, including IV fluid administration, blood pressure control, and reversal of metabolic acidosis, proved to be essential in bringing our patient’s kidney function close to the baseline and improving her overall clinical outcome.

## Conclusions

NELL-1-associated MN is remarkable in that up to one-third of patients with this disease are found to have a concomitant malignancy. Thus, it is crucial for patients who are diagnosed with NELL-1-associated MN to be evaluated for an underlying malignancy. Further research into this field could determine if certain thresholds for anti-NELL-1 antibody titers correlate with malignancy and whether there are geographical discrepancies in the target antigen causing primary MN and possible treatment options for NELL-1-specific cases of MN.
